# Acceptability and Feasibility of a Virtual Multimodal (P)Rehabilitation Programme for Gastrointestinal Cancer Patients: The PRIORITY-CONNECT 2 Pilot Randomised Controlled Trial

**DOI:** 10.1245/s10434-026-19355-0

**Published:** 2026-03-14

**Authors:** Jack Reeves, Cherry Koh, Allan Ben Smith, Helen Mohan, Sharon Carey, Stephen Smith, Thomas Poulton, Vicki Patton, Kate White, Liliana Laranjo, Mbathio Dieng, Xiaoqiu Liu, Linda Denehy, Kate Wilson, Margaret Allman-Farinelli, Phyllis Butow, Bernhard Riedel, Rachael L. Morton, Leanne Hassett, Qiang Li, Kim Delbaere, Olivia Martin, Haryana M. Dhillon, Briana Shailer, Gaynor Beardsworth, Marine Salter, Kathryn Cherry, Freya Rubie, Lauren Reece, Aveline Chan, Rihan Shahab, Olivia Dwyer, Kaylene Pring, Derek Cunningham, Kym Sheehan, Gino Iori, Rika Johnander, Claire Jeon, Nicholas Hirst, Sascha Karunaratne, Annie Zhou, Owen Hutchings, Michael Solomon, Daniel Steffens, PRIORITY-CONNECT Collaborators, Amy Cao, Nima Ahmadi, Vinna An, Nabila Ansari, Fernando Arduini, Manpreet Aulakh, Kirk Austin, Corina Behrenbruch, Nathasha Brice, Kimberley Bostock, Christopher Byrne, Ju Young Cheong, Joseph Cherng Huei Kong, David Clark, Jessica Connell, Dayan De Fontgalland, Basil D’Souza, Deshitha Gardiye Hewawasam Thuduwage, Chris Gillespie, Stephen Jancewicz, Rajni Lal, Jerome Laurence, Peter Lee, Angus Lee, Jacob McCormick, Elizabeth Murphy, Toan Pham, Carlo Pulitano, Tarik Sammour, Charbel Sandroussi, Michael Suen, Andrew Sutherland, Douglas Stupart, Howard Tang, Cheryl Tobler, Lilian Whitehead, Robert Winn, Danette Bianca Wright, Justing Yeun

**Affiliations:** 1 Surgical Outcomes Research Centre (SOuRCe), Royal Prince Alfred Hospital, Sydney, NSW Australia; 2 Discipline of Physiotherapy, Graduate School of Health, Faculty of Health, University of Technology, Sydney, NSW Australia; 3 Physiotherapy Department, Royal Prince Alfred Hospital, Sydney Local Health District, Sydney, NSW Australia; 4 RPA Institute of Academic Surgery (IAS), Royal Prince Alfred Hospital, Sydney, NSW Australia; 5 The Daffodil Centre, The University of Sydney, and Cancer Council NSW, Sydney, Australia; 6 Department of Cancer Surgery, Peter MacCallum Cancer Centre, Melbourne, VIC Australia; 7 Faculty of Medicine and Health, Sydney Nursing School, The University of Sydney, Sydney, NSW Australia; 8 Department of Colorectal Surgery, John Hunter Hospital, Sydney, NSW Australia; 9 Department of Anaesthesia, Perioperative Medicine, and Pain Medicine, Peter MacCallum Cancer Centre, Melbourne, VIC Australia; 10 Department of Critical Care, University of Melbourne, Melbourne, VIC Australia; 11 Faculty of Health Sciences, Curtin School of Nursing, Curtin University, Perth, WA Australia; 12 Faculty of Medicine and Health, Westmead Applied Research Centre, The University of Sydney, Sydney, NSW Australia; 13 NHMRC Clinical Trials Centre, The University of Sydney, Sydney, NSW Australia; 14 Biostatistics and Data Science Division, The George Institute for Global Health, University of New South Wales, Sydney, NSW Australia; 15 Department of Physiotherapy, The University of Melbourne, Melbourne, VIC Australia; 16 Department of Health Services and Implementation Science, Peter MacCallum Cancer Centre, Melbourne, VIC Australia; 17 Psycho-Oncology Co-operative Research Group (PoCoG), The University of Sydney, Sydney, NSW Australia; 18 The Sir Peter MacCallum Department of Oncology, The Department of Critical Care, The University of Melbourne, Melbourne, VIC Australia; 19 Faculty of Medicine and Health, Sydney Health Partners, Sydney School of Health Sciences, The University of Sydney, Sydney, NSW Australia; 20 Department of Allied Health, Western Sydney Local Health District, Sydney, NSW Australia; 21 Falls, Balance and Injury Research Centre, Neuroscience Research Australia (NeuRA), Sydney, NSW Australia; 22 School of Health Sciences, University of New South Wakes (UNSW), Sydney, NSW Australia; 23 Royal Prince Alfred Hospital, Sydney, NSW Australia; 24 Faculty of Science, School of Psychology, Psycho-Oncology Cooperative Research Group, University of Sydney, Sydney, NSW Australia; 25 Cancer Voices NSW, Sydney, NSW Australia; 26 Faculty of Science, School of Psychology, University of Sydney, Sydney, NSW Australia; 27 Sydney Local Health District Virtual Hospital, Sydney, NSW Australia

**Keywords:** Prehabilitation, Rehabilitation, Ttelehealth, Gastrointestinal cancer, Randomised controlled trial

## Abstract

**Background:**

Surgery is a mainstay of treatment for gastrointestinal cancers, yet it often leads to postoperative complications and poorer health outcomes. (P)rehabilitation may improve postoperative outcomes; however, optimal delivery modes remain unclear. Traditional face-to-face models may be inaccessible for patients in geographically dispersed countries. This pilot trial was designed to determine the feasibility and acceptability of a virtual multimodal (p)rehabilitation programme in patients undergoing gastrointestinal cancer surgery.

**Methods:**

The PRIORITY-CONNECT 2 Pilot trial was a multicentre, assessor-blinded, randomised controlled feasibility pilot trial. Consenting adults scheduled for gastrointestinal cancer surgery were randomised 1:1 to receive a virtual multimodal (p)rehabilitation programme (up to 6 weeks preoperatively and 3 months postoperatively) plus usual care or usual care alone. The virtual hub provided specialised multidisciplinary care by a team that included a physiotherapist, psychologist, dietitian, specialist nurse, social worker, and geriatrician. Primary outcomes included intervention feasibility (in terms of uptake, retention, and adherence) and acceptability. Secondary outcomes included postoperative complications within 30 days following surgery, health-related quality of life, and days at home within 30 and 90 days of surgery.

**Results:**

We randomised 20 participants (intervention n = 11, control n = 9). Uptake among eligible participants was 65%, retention 95%, and adherence 74%. Overall satisfaction with the programme was high; 78% of respondents reported that they were either “satisfied” or “extremely satisfied.”

**Conclusions:**

The results of this pilot trial demonstrate both feasibility and acceptability of the intervention. The full-scale PRIORITY-CONNECT 2 Trial will determine the effectiveness of a virtual multimodal (p)rehabilitation programme for patients undergoing colorectal cancer surgery.

*Trial registration*

This trial was registered prospectively with the National Library of Medicine ClinicalTrials.gov Registry (NCT06212700) on 8th January 2024.

Gastrointestinal cancers are responsible for nearly a third of all cancer-related deaths.^1^ Surgical resection, with or without neoadjuvant chemo- and/or radiation therapy, remains a mainstay of treatment.^2^ Postoperative complications following gastrointestinal cancer surgery are common and can lead to negative outcomes, such as increased length of hospital stay, health care costs, and mortality.^3,4^ Individualised perioperative assessment and targeted interventions may help to address known risk factors that contribute to higher postoperative complications.^5^

Prehabilitation is defined as a process from diagnosis to surgery, during which one or more interventions (e.g., exercise, nutrition, psychological support, or respiratory training) are delivered to enhance functional capacity and physiological reserve to allow patients to better tolerate surgical stress and recover more efficiently.^6^ A recent umbrella review found that despite high risk of bias and substantial heterogeneity across studies, prehabilitation supported functional recovery (moderate certainty), reduced postoperative complications and length of hospital stay, and increased chance of discharge to home (low to very low certainty).^7^ Postoperative rehabilitation interventions have also been shown to improve patient outcomes. An overview of systematic reviews (n = 60 reviews, including 761 randomised controlled trials) in those with colorectal cancer reported moderate-quality evidence supporting exercise-based rehabilitation to improve physical fitness and quality of life.^8^

The evidence base for (p)rehabilitation interventions (i.e., combined prehabilitation and rehabilitation approaches) is predominantly derived from face-to-face delivery models, which may not be accessible within geographically disparate countries. This traditional approach to care delivery may introduce selection bias and inequities, because patients living far from specialised (p)rehabilitation centres are often excluded from studies or unable to access these services. Virtual delivery models, such as via telehealth platforms, may offer a more equitable and scalable solution for delivering (p)rehabilitation programmes to geographically dispersed populations, potentially improving access while maintaining intervention fidelity.

The PRIORITY-CONNECT 2 pilot trial was undertaken to assess feasibility and acceptability prior to a large-scale hybrid Type I randomised controlled trial evaluating effectiveness and implementation outcomes of a virtual multimodal hub delivering a prehabilitation-rehabilitation programme. The primary aim of this pilot trial was to determine the feasibility (i.e., uptake, retention, adherence) and acceptability of a virtual multimodal programme delivered during the preoperative and postoperative period for patients undergoing major gastrointestinal cancer surgery. The secondary aims were to obtain pilot data on the likely difference in key outcomes including postoperative complications within 30 days following surgery, health-related quality of life, and days at home within 30 and 90 days of surgery.

## Methods

### Study Design

The PRIORITY-CONNECT 2 Pilot trial was a multicentre, assessor-blinded, randomised controlled feasibility trial. This pilot trial report adheres to the Consolidated Standards for Reporting Trials (CONSORT) Guidelines and the previously published protocol.^9,10^ This article focuses on feasibility, acceptability, and preliminary clinical outcomes. Qualitative and healthcare expenditure outcomes described in the published protocol will be reported in subsequent articles. Participating sites included two hospitals in Sydney, Australia, which are nationally recognised for surgical management of patients with gastrointestinal cancer: Royal Prince Alfred (lead site) and Chris O’Brien Lifehouse (satellite site). This pilot trial was prospectively registered with the National Library of Medicine (ClinicalTrials.gov) Registry (NCT06212700). The study obtained ethical approval from the Sydney Local Health District Human Research and Ethics Committee – Royal Prince Alfred Hospital (2023/ETH02359).

### Participants

Participants were undergoing major gastrointestinal cancer surgery. To be included, participants had to be adults ≥18 years of age, scheduled for either liver, pancreas, oesophagus, gastric, or colorectal cancer resection with curative intent. Participants were also required to have a consultation with a gastrointestinal cancer surgeon at least 1 week in advance of surgery to be eligible. Exclusion criteria were cognitive impairment rendering people unable to provide informed consent as well as inability to access a smart device (including mobile phone, tablet, laptop, or desk computer with camera) or no internet connection. Those undergoing neoadjuvant therapy (such as chemo-/radiation therapy) were included. Patients were identified and screened for eligibility by a multidisciplinary team which included surgeons, anaesthetists, clinical nurse consultants, and research officers. Those who met the eligibility criteria and provided informed consent were included in the study.

### Randomisation and Blinding

Immediately prior to randomisation, eligible and consenting participants underwent baseline assessment. Participants were then randomised (1:1 using blocks of 2 and 4) to either the virtual multimodal hub plus usual care (intervention group) or usual care alone (control group) via a Research Electronic Data Capture (REDCap) database using an a priori central secure randomisation service. This was developed by the trial biostatistician and uploaded to REDCap by an independent research officer not involved in the pilot trial. Randomisation was stratified by hospital to ensure balance of treatment assignments. Treatment allocation was concealed from those involved in undertaking baseline assessment and randomisation of participants. Most trial personnel, including the treating surgeon, participant assessors, and the biostatistician, were blind to which group participants were allocated. Participants receiving, and clinicians delivering, the intervention were unable to be blinded due to the nature of the intervention.

### Intervention

Those randomised to the intervention accessed a virtual multimodal hub, which delivered specialised multidisciplinary care by a team, including a physiotherapist, psychologist, dietitian, specialist nurse, social worker, and geriatrician. The geriatrician’s role was to provide comprehensive geriatric assessment to participants ≥75 years of age. The virtual multimodal hub intervention was delivered via videoconferencing (Microsoft Teams) to the participant’s chosen device both preoperatively (within 1–6 weeks prior to surgery) and postoperatively (up to 3 months following surgery). Given that surgery can often be scheduled soon after surgical consultation, a minimum preoperative period of 1 week was chosen to ensure an adequate number of eligible patients could participate in the pilot trial. Included participants who were only able to receive the preoperative aspect of the intervention for a short duration could still benefit from the postoperative aspect.

The purpose of the virtual multimodal hub was to (1) address modifiable risk burden preoperatively (e.g., physical deconditioning, malnutrition); (2) prepare participants for surgery (including expectation setting); and (3) provide support to minimise decline in physiological and psychological function associated with preoperative and postoperative neoadjuvant treatments and surgery. Postoperative rehabilitation focused on interventions to recover activities of daily living, occupational tasks, and recreational activities. Multimodal interventions were individualised in terms of frequency, intensity, time, type, volume, and progression, based on a comprehensive assessment at baseline and postdischarge. This included an assessment of participants’ physical, nutritional, and psychological status as well as their comorbidities and overall health status. Participants in the rehabilitation phase (i.e., postoperative) who met their pretargeted treatment goals (co-developed by healthcare professionals and participants) were discharged, even if they remained within the 3-month postoperative intervention period. A full description of the intervention according to the Template for Intervention Description and Replication Checklist (TIDieR) can be found in the published protocol.^10,11^

### Control

Those randomised to the control group received usual care according to their normal healthcare team. This involved advice on smoking cessation, reduction of alcohol intake, general advice on exercise, nutritional counselling, and medical optimisation during preoperative anaesthetic visits. Those in the control group were asked to maintain their usual daily activities. Any external prehabilitation, rehabilitation, or advanced care services provided outside of the pilot trial were recorded for both groups.

### Outcome Measures

The primary aim of the PRIORITY-CONNECT 2 Pilot trial was to assess the feasibility and acceptability of the virtual multimodal hub. Feasibility was determined by three criteria: (1) uptake: the proportion of eligible participants recruited to the trial; (2) retention rate: defined as the percentage of participants who completed the trial; (3) frequency and adherence rates: defined as the number and percentage of planned sessions attended by participants randomised to the intervention. Adherence was monitored using multidisciplinary session attendance records and participant diaries. To assess acceptability, participants were asked to complete a previously used 11-question Acceptability and Satisfaction Survey using a 5-point Likert scale assessing aspects of the programme.^12,13^

Trial outcomes were assessed at baseline (between 1-6 weeks before scheduled surgery), 1 to 2 days prior to surgery, day of discharge from hospital, and 3 months following index surgery. All outcomes were collected by trained trial personnel blinded to participant group allocation. Secondary outcomes were collected to explore preliminary efficacy signals rather than to evaluate effectiveness. Postoperative complications within 30 days of surgery, defined as any deviation from the normal postoperative course as according to the Clavien-Dindo Classification, were recorded.^14^ Health-related quality of life (QoL) was assessed using the European Organisation for Research and Treatment of Cancer (EORTC) 30-item QoL in cancer patients questionnaire (QLQ-C30), the 25-item QoL in oesophago-gastric cancer patients (QLQ-OG25), and the 29-item QoL in colorectal cancer patients (QLQ-CR29) scales.^15–17^ Assessment of QoL occurred at timepoints listed above and at 1 month following index surgery. Number of days at home and out of hospital, within 30 (DAH30) and 90 (DAH90) days of surgery were also collected.^18^

### Sample Size

This pilot trial aimed to evaluate feasibility and acceptability, and as such, a sample size calculation to power the study to evaluate effectiveness was not required. A total of 20 participants (aiming for 10 in each arm) was determined based on the feasibility of recruitment within the study timeline.

### Statistical Analysis

A blinded intention-to-treat analysis was conducted by using IBM SPSS Version 29 (SPSS Inc., Chicago, IL). Statistical analysis evaluated feasibility and acceptability outcomes by descriptively reporting the number and percentage of participants who engaged with the programme at various stages as well as primary and secondary outcomes.

## Results

Participants were both screened and recruited between February and June 2024. Baseline characteristics of included participants in each group are reported in Table 1. Participants presented with a range of gastrointestinal cancers including colorectal, pancreatic, pseudomyxoma peritonei, ovarian, cervical, and gastrointestinal stromal tumours.

**Table 1:** Baseline characteristics and surgical outcomes

Characteristics	Intervention	Control	Overall
	N = 11 (%)	N = 9 (%)	N = 20 (%)
Age, years, median (IQR)	51.0 (47 to 51)	62.0 (57 to 68)	53.0 (50–67)
Sex (males)	5 (46)	3 (33)	8 (40)
Country of birth (Australia)	10 (91)	5 (63)^a^	15 (79)^b^
Health insurance (Private)	8 (73)	9 (100)	17 (85)
Currently employed	9 (82)	6 (67)	15 (75)
Highest level of education			
Leaving certificate or below	2 (18)	3 (33)	5 (25)
Diploma or higher	9 (82)	6 (67)	15 (75)
Annual personal income (AUD, gross)			
Nil income	1 (9)	0 (0)^a^	1 (5)^b^
$1–65,000	2 (18)	3 (38)^a^	5 (26)^b^
$65,000 or more	8 (73)	5 (63)^a^	13 (68)^b^
Body mass index (BMI) kg/m^2^, median (IQR)	29 (27–30)	26 (22–28)^a^	27 (25–29)^b^
Smoking			
Former smoker	5 (46)	4 (44)	9 (45)
Never smoked	6 (55)	5 (56)	11 (55)
Neoadjuvant treatment (yes)	4 (36)	2 (22)	6 (30)
Comorbidities (yes)	7 (64)	7 (78)	14 (70)
Primary tumour			
Colorectal	7 (64)	2 (22)	9 (45)
Pancreatic	2 (18)	3 (33)	5 (25)
Pseudomyxoma peritoneii	0 (0)	2 (22)	2 (10)
Gastrointestinal stromal tumours	0 (0)	1 (11)	1 (5)
Phaeochromocytoma	0 (0)	1 (11)	1 (5)
Ovarian	1 (9)	0 (0)	1 (5)
Cervical	1 (9)	0 (0)	1 (5)
Primary procedure			
Cytoreductive surgery	3 (27)	3 (33)	6 (30)
Colectomy	3 (27)	1 (11)	4 (20)
Pancreatectomy	1 (9)	3 (33)	4 (20)
Pelvic exenteration	2 (18)	0 (0)	2 (10)
Liver wedge resection	1 (9)	0 (0)	1 (5)
Gastrectomy	0 (0)	1 (11)	1 (5)
Pancreaticoduodenectomy	1 (9)	0 (0)	1 (5)
Adrenalectomy	0 (0)	1 (11)	1 (5)
Blood transfusion (units), median (IQR)	2 (2–14)	14 (11–25)	11 (3–23)
Intraoperative complication (yes)	1 (9)	0 (0)	1 (5)
Length of operation (hours), median (IQR)	8 (3–8)	4 (3–9)	5 (3 to 8)
Length of ICU stay (days), median (IQR)	2 (2–4)	2 (1–4)	2 (2 to 4)
Length of hospital stay (days), median (IQR)	11 (10–22)	17 (12–17)	14 (10–20)
Clear surgical margin (R0/CC0)	6 (100)^c^	4 (80)^d^	10 (91)^e^
Discharge destination (Home)	10 (91)	8 (89)	18 (90)
Hospital re-admission (90-days, yes)	3 (27)	1 (11)	4 (20)
Mortality (90-days, yes)	0 (0)	0 (0.0)	0 (0)

^a^N = 8; ^b^N = 19; ^c^N = 6; ^d^N = 6; ^e^N = 11. All data are presented as frequency (%) or median (IQR) or unless stated otherwise

### Feasibility

**Fig. 1:**
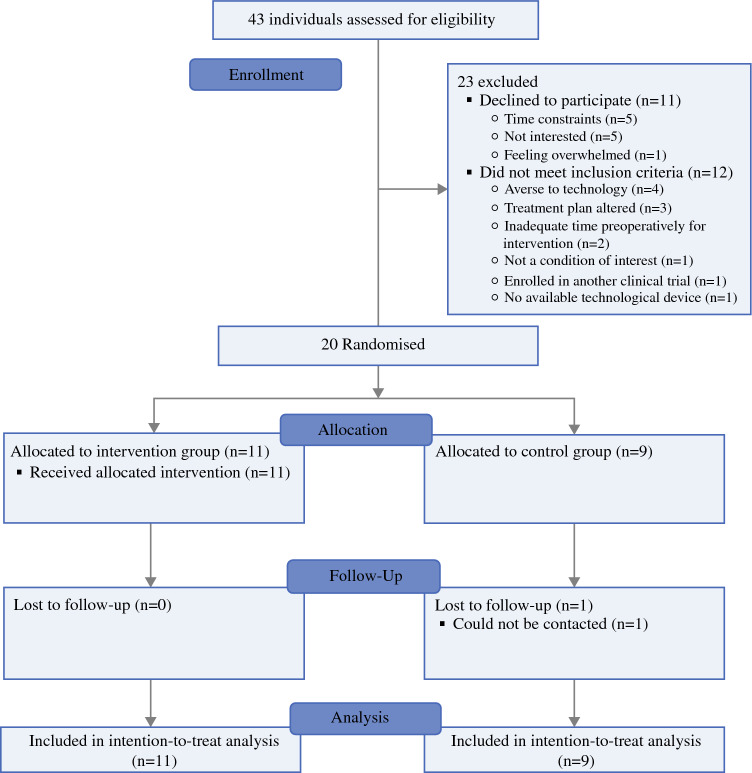
Consort diagram

Participant flow through the study is shown in Fig. 1. Forty-three individuals were screened for eligibility, and 31 met the inclusion criteria. Of those eligible for inclusion, 11 declined to participate, and the remaining 20 (65%) were randomised to intervention (n = 11) or control groups (n = 9). Of the 20 participants randomised, 19 (95%) completed the trial, including 11 (100%) from the intervention group and eight (89%) from the control group. Frequency and adherence rates are reported in Table 2. Adherence to scheduled sessions was high within the intervention group. Of 105 total scheduled sessions, 77 (74%) were attended, including 31 of 39 (80%) preoperative sessions and 46 of 66 (70%) postoperative sessions. No major technical issues were identified, as discussed in a separate in-depth qualitative study.

**Table 2:** Frequency and adherence rates of the virtual multimodal hub (N = 11)

	Preoperative sessions scheduledN = 39 (%)	Postoperative sessions scheduledN = 66 (%)	Total sessions scheduledN = 105 (%)
Adherence rate	31 (80)	46 (70)	77 (73)
Reasons for nonattendance			
Did not attend or respond to follow up	2 (25)	6 (30)	8 (29)
Conflicting work commitments	0 (0)	4 (20)	4 (14)
Feeling unwell	1 (12)	4 (20)	5 (18)
Rescheduled	0 (0)	3 (15)	3 (10)
Conflicting personal plans	1 (12)	1 (5)	2 (7)
Conflicting treatment consultation	3 (38)	0(0)	3 (10)
Cancer-related complication	1 (12)	0 (0)	1 (4)
Treatment-related complication	0 (0)	1 (5)	1 (4)
Self-discharge	0 (0)	1 (5)	1 (4)

N = 11 (intervention only). All data are presented as Frequency (%) unless stated otherwise

Data related to the completion of questionnaires are reported in Table 3. In total, 80 questionnaires were administered and 74 completed across 20 participants. No participants required assistance when completing questionnaires. Questionnaires were predominantly completed online with the remaining few completed via telephone. The most common reason for incomplete questionnaires was an uncontactable participant.

**Table 3:** Questionnaire completion information

	Intervention questionnaires administered	Control questionnaires administered	Total questionnaires administered
	N = 44 (%)	N = 36 (%)	N = 80 (%)
Proportion of participants who required assistance to complete the questionnaire	0 (0)	0 (0)	0 (0)
Method of questionnaire administration			
Face-to-face	0 (0)	0 (0)	0 (0)
By telephone	1 (2)	2 (6)	3 (4)
Online	43 (98)	34 (94)	77 (96)
Mail	0 (0)	0 (0)	0 (0)
Most appropriate reason for noncompletion of the questionnaire			
Patient received the questionnaire but did not return them	1 (2)	0 (0)	1 (1)
Patient refused to complete questionnaire	0 (0)	0 (0)	0 (0)
Unable to contact patient	1 (2)	4 (11)	5 (6)

All data are presented as frequency (%) unless stated otherwise

### Acceptability

Results of the Acceptability and Satisfaction Survey can be seen in Table 4. Overall satisfaction with the programme was high, with 78% of respondents reporting they were either “satisfied” or “extremely satisfied” with the intervention. Additionally, 90% of respondents were confident they could follow the advice provided (assessed as ≥7/10 on a 10-point scale).

**Table 4:** Results of the Acceptability and Satisfaction Survey of those delivered the virtual multimodal hub (N = 10)^*^

Variable	Strongly disagree	Disagree	Neutral	Agree	Strongly agree
I was satisfied with the advice received by my treating clinicians	0 (0)	0 (0)	1 (10)	4 (40)	5 (50)
I was satisfied with the amount of advice received	0 (0)	0 (0)	0 (0)	5 (50)	5 (50)
My relatives were supporting my participation in the virtual multimodal hub program	0 (0)	0 (0)	2 (20)	5 (50)	3 (30)
The recommended advice was easy to understand and follow	0 (0)	0 (0)	0 (0)	5 (50)	5 (50)
I had no problems in accessing the virtual multimodal hub to attend the sessions	0 (0)	0 (0)	0 (0)	6 (60)	4 (40)
The duration of the sessions was just right for me	0 (0)	0 (0)	2 (20)	4 (40)	4 (40)
The intensity of the sessions was just right for me	0 (0)	0 (0)	1 (10)	4 (40)	5 (50)
I would recommend the virtual multimodal hub to a friend	0 (0)	0 (0)	2 (20)	2 (20)	6 (60)
The virtual multimodal hub covered all my needs	0 (0)	0 (0)	2 (20)	3 (30)	5 (50)
My participation in the virtual multimodal hub was influenced by the advice of my surgeon	0 (0)	3 (30)	2 (20)	2 (20)	3 (30)
The virtual multimodal hub helped me to understand the importance of preparing for surgery	0 (0)	0 (0)	2 (20)	5 (50)	3 (30)
Overall satisfaction^**^					
	Extremely dissatisfied	Dissatisfied	Neutral	Satisfied	Extremely satisfied
Which of the following categories best describes your satisfaction with study treatment	0 (0)	0 (0)	2 (22)	2 (22)	5 (56)
Confidence	Not confident 0-2	3–4	5–6	7–8	Extremely confident 9-10
How confident you were in your ability to follow the advice that was given	0 (0)	0 (0)	1 (10)	4 (40)	5 (50)

^*^There was one nonresponder to this questionnaire within the intervention group. ^**^N = 9. All data are presented as frequency (%) unless stated otherwise

### Secondary Outcomes

Secondary outcomes are reported in Table 5. Twelve (60%) participants experienced complications within the first 30 days following surgery; eight (73%) in the intervention group and four (44%) in the control group. In terms of days at home within the first 30 days of surgery (DAH30), the intervention group had a median (interquartile range [IQR]) of 19.0 days (4.5–21.5) and the control group 13.0 days (13.0–22.0). Days at home within 90 days of surgery (DAH90) in the intervention group was 79 days (58.5–81.5) and in the control group was 73 days (73.0–82.0). Further patient-reported outcomes and physical measures assessed are reported in Table 6. These included pain, exercise capacity, self-reported physical activity, nutritional status, anxiety, depression, and self-efficacy. No adverse events were reported directly due to participation in the trial.

**Table 5:** Descriptive table of secondary outcome measures

Variable	Intervention	Control	Total
	N = 11 (%)	N = 9 (%)	N = 20 (%)
Proportion of participants developing complications within 30 days after surgery	8 (73)	4 (44)	12 (60)
Highest Clavien-Dindo classification (30 day)			
Minor complication (Grade I-II)	4 (36)	1 (11)	5 (25)
Major complication (Grade III-V)	4 (36)	3 (33)	7 (35)
Days at home and out of hospital			
DAH-30, median (IQR)	19.0 (5–22)	13.0 (13–22)	16.5 (7–22)
DAH-90, median (IQR)	79.0 (59–82)	73.0 (73–82)	76.5 (67–82)
Quality of life (EORTC QLQ-C30)			
Global health status, total	75.0 (70–85)^a^	65.0 (54–81)^b^	75.0 (60–85)^c^
Baseline	80.0 (75–85)	72.5 (63–86)^d^	80.0 (73–85)^e^
Days before surgery	85.0 (75–89)^f^	75.0 (69–86)	80.0 (75–89)^g^
Discharge	60.0 (53–70)	52.5 (48–63)^d^	55.0 (50–70)^e^
3 Months	77.5 (70–85)^f^	60.0 (50–71)	70.0 (61–85)^g^
Summary score	86.0 (79–91)^a^	78.4 (69–94)^b^	83.4 (75–92)^c^
Baseline	89.2 (83–92)	85.4 (75–96)^d^	87.4 (81–94)^e^
Days before surgery	89.8 (86–96)^f^	83.3 (76–96)	89.2 (81–96)^g^
Discharge	77.2 (37–81)	68.2 (61–75)^d^	70.6 (50–80)^e^
3 Months	85.8 (82–91)^f^	82.9 (70–95)	85.8 (79–93)^g^

One patient was a nonresponder to questionnaires within the control group. ^a^N = 42; ^b^N = 32; ^c^N = 74; ^d^N = 8; ^e^N = 19; ^f^N = 10; ^g^N = 18. All data are presented as median (IQR) or frequency (%) unless stated otherwise. The Global health status and Summary scores of the EORTC QLQ-C30 range from 0 to 100; a higher score represents a better quality of life

**Table 6:** Descriptive table of assessment measures

Outcomes	Intervention	Control	Total
	N = 11 (range)	N = 9 (range)^a^	N = 20 (range)^b^
Pain (NPRS)			
Baseline	2.0 (1–6)	1.5 (0–4.5)	2.0 (0–6)
Days before surgery	1.5 (0–3)^c^	0.0 (0–4.3)	1.0 (0–4)^d^
Discharge	4.0 (1–4)	3.5 (1–6.3)	4.0 (1–5)
3 months	2.0 (0–3)^c^	0.0 (0–4.0)	1.0 (0–3)^d^
Exercise capacity (1MSTS)			
Baseline	27 (25–34)^c^	26 (24–62)	26 (24–35)^d^
Days before surgery	32 (26–36)^e^	24 (22–31)^f^	30 (23–36)^g^
Discharge	24 (2–27)^a^	23 (18–43)^e^	23 (19–27)^h^
3 months	27 (22–32)^c^	24 (21–28)^i^	26 (21–31)^j^
Self-reported physical activity (IPAQ)			
Baseline MET minute/week	2,475 (132–17,456)	1,992 (173–23,102)	2,475 (132–21,910)
Days before surgery MET minute/week	4,524 (361–24,426)^c^	9,489 (446–22,523)	4,524 (347–24,426)^d^
Discharge MET minute/week	792 (269–2,856)	314 (124–2,945)	396 (215–2,856)
3 months MET minute/week	2 067 (862–4,108)^c^	5,282 (1,238–10,928)	2,520 (862–5,529)^d^
Nutritional status (PG-SGA)			
Baseline	0 (0–0)	0 (0–2)	0 (0–0)
Days before surgery	0 (0–0)^c^	0 (0–2)	0 (0–0)^d^
Discharge	4 (3–4)	3 (1–4)	3 (3–4)
3 months	0 (0–1)^c^	0 (0–0)	0 (0–0)^d^
Anxiety (GAD-7)			
Baseline	3.0 (0–5)	0.5 (0–3)	1 (0–4)
Days before surgery	0.0 (0–3)^c^	0.5 (0–3)	0.5 (0–3)^d^
Discharge	2.0 (0–4)	0.0 (0–1)	0.5 (0–3)
3 months	0.0 (0–1)^c^	0.0 (0–3)	0.5 (0–1)^d^
Depression (PHQ-9)			
Baseline	1.0 (0–4)	2.0 (0–6)	2.0 (0–5)
Days before surgery	1.0 (0–3)^c^	2.0 (0–3)	1.0 (0–3)^d^
Discharge	6.0 (3–11)	4.0 (1–5)	4.5 (2–7)
3 months	1.5 (1–4)^c^	2.5 (0–5)	2.0 (0–4)^d^
Self-efficacy (GSE)			
3 months	33 (31–38)^c^	35 (29–40)^i^	33 (30–38)^j^
No. adverse events (AE)	0 (0)	0 (0)	0 (0)

One patient was a nonresponder to questionnaires within the control group. ^a^N = 8; ^b^N = 19; ^c^N = 10; ^d^N = 18; ^e^N = 7; ^f^N = 5; ^g^N = 12; ^h^N = 15; ^i^N = 6; ^j^N = 16. All data are presented as median (IQR) or frequency (%) unless stated otherwise. *NPRS* Numerical Pain Rating Scale, with 0 indicating no pain, up to 10 indicating worst possible pain. *1MSTS* 1 minute sit-to-stand test. *IPAQ* International Physical Activity Questionnaire; *MET* Metabolic Equivalent of Task; PG-SGA weight score; 0–1 indicates well nourished, 2–3 is moderately malnourished or suspected malnourishment, and 3–5 represents severe malnourishment. GAD-7 score; 0–4 indicates minimal anxiety, 5–9 mild anxiety, 10–14 moderate anxiety, and 15–21 severe anxiety. PHQ-9 score; 1–4 indicates minimal depression, 5–9 mild depression, 10–14 moderate depression, 15–19 moderately severe depression, 20–27 severe depression. GSE total score ranges between 10 and 40; a higher score indicates more self-efficacy

The number and type of complications within 30 days of surgery, including Clavien-Dindo classification (minor [grade I-II] or major [grade III-V]), are reported in (Table 7). There was a total of 22 complications among the 12 participants who experienced a complication: 13 from the intervention group and nine from the control group. Of the 22 complications, 15 (68%) were minor and seven (32%) were major. Complications were surgical (n = 7), respiratory (n = 5), infection-related (n = 3), haematological (n = 2), renal (n = 1), pain-related (n = 1), or other (n = 3).

**Table 7:** 30-day postoperative complications

	Intervention (N = 11)	Control (N = 9)	Total (N = 20)
Respiratory complications	2 (18)	3 (33)	5 (25)
Minor (Clavien-Dindo grade I-II)	1 (9)	1 (11)	2 (10)
Major (Clavien-Dindo grade III-V)	1 (9)	2 (22)	3 (15)
Infectious complications	2 (18)	1 (11)	3 (15)
Minor (Clavien-Dindo grade I-II)	1 (9)	1 (11)	2 (10)
Major (Clavien-Dindo grade III-V)	1 (9)	0 (0)	1 (5)
Cardiovascular complications	0 (0)	0 (0)	0 (0)
Minor (Clavien-Dindo grade I-II)	0 (0)	0 (0)	0 (0)
Major (Clavien-Dindo grade III-V)	0 (0)	0 (0)	0 (0)
Renal complications	1 (9)	0 (0)	1 (5)
Minor (Clavien-Dindo grade I-II)	1 (9)	0 (0)	1 (5)
Major (Clavien-Dindo grade III-V)	0 (0)	0 (0)	0 (0)
Gastrointestinal complications	0 (0)	0 (0)	0 (0)
Minor (Clavien-Dindo grade I-II)	0 (0)	0 (0)	0 (0)
Major (Clavien-Dindo grade III-V)	0 (0)	0 (0)	0 (0)
Surgical complications	5 (46)	2 (22)	7 (35)
Minor (Clavien-Dindo grade I-II)	3 (27)	1 (11)	4 (20)
Major (Clavien-Dindo grade III-V)	4 (36)	1 (11)	5 (25)
Neurological complications	0 (0)	0 (0)	0 (0)
Minor (Clavien-Dindo grade I-II)	0 (0)	0 (0)	0 (0)
Major (Clavien-Dindo grade III-V)	0 (0)	0 (0)	0 (0)
Haematological complications	0 (0)	2 (22)	2 (10)
Minor (Clavien-Dindo grade I-II)	0 (0)	2 (22)	2 (10)
Major (Clavien-Dindo grade III-V)	0 (0)	0 (0)	0 (0)
Wound complications	0 (0)	0 (0)	0 (0)
Minor (Clavien-Dindo grade I-II)	0 (0)	0 (0)	0 (0)
Major (Clavien-Dindo grade III-V)	0 (0)	0 (0)	0 (0)
Pain complications	1 (9)	0 (0)	1 (5)
Minor (Clavien-Dindo grade I-II)	1 (9)	0 (0)	1(5)
Major (Clavien-Dindo grade III-V)	0 (0)	0 (0)	0 (0)
Other complications	2 (18)	1 (11)	3 (15)
Minor (Clavien-Dindo grade I-II)	2 (18)	1 (11)	3 (15)
Major (Clavien-Dindo grade III-V)	0 (0)	0 (0)	0 (0)

All data presented as Frequency (%) unless stated otherwise.

## Discussion

The PRIORITY-CONNECT 2 Pilot trial evaluated feasibility and acceptability of a virtual multimodal (p)rehabilitation programme for people undergoing gastrointestinal cancer surgery. The trial was deemed feasible based on participant uptake, retention, and adherence, and participants found the intervention acceptable. This pilot randomised controlled trial generated valuable preliminary data that has informed trial design, target population, and delivery of the virtual multimodal hub.

Feasibility and acceptability results from this trial, using a virtual multimodal approach, are similar to pilot trials evaluating in-person approaches. The PEPA pilot trial evaluated feasibility and acceptability of a centre-based preoperative exercise programme in those undergoing pelvic exenteration or cytoreductive surgery. PEPA demonstrated a participant retention rate of 91% (current study 95%), study uptake rate by eligible individuals of 85% (current study 65%), and intervention adherence rate of 93% (current study 74%).^19^ A further study evaluated feasibility of an in-person multimodal prehabilitation programme for women undergoing cytoreductive surgery for advanced ovarian cancer. They reported an overall adherence rate of 80% to their multimodal intervention, which similarly involved exercise training, nutritional optimisation, and psychological support.^20^ These comparable results suggest that transitioning to a virtual model does not compromise engagement or retention and may broaden access for patients living remotely.

A range of secondary outcomes related to the full-scale trial were collected. Notably, there were differences in the mean age and complexity of oncological surgery between the intervention and control groups, which were not balanced by randomisation due to the small sample size in this feasibility and acceptability trial. Consequently, full-scale trials should consider stratification for surgical complexity to ensure balance between groups in their randomisation approach. We hypothesise with a greater sample size, the intervention will reduce the risk of 30-day postoperative complications, supported by previous trials assessing preoperative multimodal interventions.^21–23^

Preoperative assessment is often focused on one domain in isolation rather than multiple domains (i.e., physical, psychological, and nutritional).^24^ A strength of this trial was its use of a multidisciplinary team of health professionals (including physiotherapist, psychologist, dietitian, specialist nurse, social worker, and geriatrician) to assess participants and deliver a tailored intervention. An initial concern of utilising such a comprehensive multidisciplinary approach was that patients may have been burdened by several appointments with healthcare professionals in addition to their preexisting commitments and therefore have poorer treatment satisfaction. Fortunately, this was not the case as participants demonstrated satisfaction with the intervention based on survey results. Additionally, whilst unimodal interventions (i.e., exercise-based alone) are likely to reduce postoperative complications and length of hospital stay in those undergoing cancer surgery prehabilitation,^25^ a systematic review comparing unimodal interventions with multimodal interventions (in oesophagogastric surgery) has demonstrated multimodal interventions are more effective at decreasing postoperative complication rate and severity.^26^

This pilot trial has limitations. Feasibility and acceptability data presented here are specific to the context in which it was conducted, i.e., two large cancer surgical services in Sydney, Australia, and involving only English-speaking participants. Whilst this feasibility data supports the next phase of the virtual multimodal hub (i.e., a full-scale effectiveness-implementation randomised controlled trial) it may be less generalisable to other settings. Future studies should examine the effectiveness of virtual multimodal programmes in reducing postoperative complications and improving quality of life for people with gastrointestinal cancers. Whilst there is evidence in favour of centre-based multimodal prehabilitation and rehabilitation interventions in those undergoing cancer surgery, understanding the effectiveness of virtual programmes may offer greater patient access and allow integration of such programmes into preoperative care pathways.

### Patient and Public Involvement

This research program has benefited from more than a decade of continuous and meaningful involvement from highly experienced consumer representatives, ensuring that patient perspectives have shaped every stage of development. Consumers have been embedded as core partners from the earliest phases of concept development through to study design, participant materials, recruitment approaches, intervention refinement, and reporting. Their lived experience and deep understanding of gastrointestinal cancer care strengthened the relevance, accessibility and acceptability of the virtual multimodal (p)rehabilitation model. Consumer representatives also contributed to interpretation of pilot findings and advised on strategies to optimise implementation and scale-up for the full PRIORITY-CONNECT 2 trial. Their long-standing partnership has ensured that the trial remains firmly grounded in the needs, values, and priorities of the patients it seeks to support.

## Conclusions

The PRIORITY-CONNECT 2 Pilot Trial was deemed both feasible and acceptable and therefore will proceed to a phase-3 multicentre hybrid type 1 effectiveness-implementation randomised controlled trial, the PRIORITY-CONNECT 2 Trial. This full-scale trial aims to determine the effectiveness of a virtual multimodal hub delivering a (p)rehabilitation programme for reducing postoperative complications following colorectal cancer surgery.
